# Anxiety and depression among healthcare workers during the COVID-19 pandemic in Khujand, Tajikistan

**DOI:** 10.3389/fpubh.2026.1875324

**Published:** 2026-06-24

**Authors:** Jamila Silemonshoeva, Roberta Horth, Zulfiya Tilloeva, Amirzoda Abduholik, Edmond F Maes, Shalkar Adambekov, Alexander J. Millman, Dilyara Nabirova

**Affiliations:** 1 Central Asia Advanced Field Epidemiology Training Program, Dushanbe, Tajikistan; 2 Asfendiyarov Kazakh National Medical University, Almaty, Kazakhstan; 3 Center of State Sanitary and Epidemiological Service of Sughd Region, Khujand, Tajikistan; 4 United States Centers for Disease Control and Prevention, Central Asia Office, Almaty, Kazakhstan; 5 State Institution "Municipal Disinfection Station", Dushanbe, Tajikistan; 6 Ministry of Health and Social Protection of Population of the Republic of Tajikistan, Dushanbe, Tajikistan; 7 Rollins School of Public Health, Emory University, Atlanta, GA, United States

**Keywords:** COVID-19, healthcare workers, mental health, pandemic, Tajikistan

## Abstract

**Background:**

The COVID-19 pandemic strained healthcare worker (HCW) mental health worldwide. In Tajikistan, limited data on HCW mental health limit understanding of the pandemic’s effect on HCWs. We assessed the prevalence of anxiety and depression and examined associated occupational and psychosocial factors among HCWs who provided care during the COVID-19 pandemic from March 2020 to December 2021.

**Methods:**

We conducted a cross-sectional survey of HCWs from three hospitals and three polyclinics in Khujand, Tajikistan, during July–August 2022. Participants were asked to recall their experiences from March 2020 through December 2021. Using systematic sampling, 400 HCWs who had provided care to patients with COVID-19 were interviewed in person. Moderate-to-severe anxiety and depression symptoms were assessed using the Hospital Anxiety and Depression Scale (cut-off scores of ≥11). Associations with anxiety and depression were examined using multivariable mixed-effects models adjusting for hospital clustering.

**Results:**

Among 400 participants, 69% had depression, 26% anxiety, and 17% both. Most participants were female (82%), nurses (67%), and had more than 10 years of work experience (51%). Anxiety was independently associated with female sex [adjusted prevalence odds ratio (aPOR) = 3.2; 95% confidence interval (CI): 1.5–6.6], work fatigue (aPOR = 2.4; 95% CI: 1.0–5.7), prior COVID-19 (aPOR = 2.2; 95% CI: 1.3–3.9), working ≥12-h shifts vs. less (aPOR = 1.9; 95% CI: 1.1–3.2), and family separation during the pandemic (aPOR = 1.8; 95% CI: 1.0–3.2). Depression was associated with having had severe COVID-19 vs. mild or none (aPOR = 4.0; 95% CI: 1.4–11.8), nurse vs. physician role (aPOR = 3.5; 95% CI: 1.7–7.2), and work-related social avoidance vs. none (aPOR = 1.6; 95% CI: 1.0–2.6).

**Conclusion:**

HCWs in Tajikistan experienced a substantial burden of anxiety and depression during the COVID-19 pandemic. These findings were used to inform mental health trainings, screening programs, and workplace interventions including counseling access, respite programs, and improved staffing patterns to support healthcare workers experiencing anxiety and depression.

## Introduction

The COVID-19 pandemic placed unprecedented strain on healthcare systems worldwide, exposing healthcare workers to elevated risks of infection as well as substantial mental health consequences (1, 2). During the first year of the pandemic, global estimates suggested that between 80,000 and 180,000 healthcare workers died from COVID-19 globally, while many others experienced anxiety, depression, and burnout (3). Systematic reviews have reported pooled prevalence estimates of approximately 22% for anxiety and depression among healthcare workers during the pandemic (4), outcomes that can be understood through the Job Demands–Resources model, which explains how high job demands increase anxiety and depression while job resources support worker well-being (5).

The mental health burden among HCWs evolved over the course of the pandemic. During the acute phase in 2020, responses were characterized by fear of infection, moral distress, and crisis-level workload. As the pandemic progressed through 2021–2022, evidence from multiple countries documented a transition toward chronic occupational burnout, organizational fatigue, and cumulative psychological exhaustion (6). These later-phase effects were often compounded in low- and middle-income countries, where healthcare infrastructure was more strained, PPE supplies were more limited, mental health services were less available, and staff-to-patient ratios were lower (7). Known sociodemographic correlates of mental health distress among HCWs include female sex, nursing roles, younger age, and lower seniority—factors consistently identified across pandemic-era studies (4, 8).

Tajikistan reported its first confirmed COVID-19 case in April 2020. By mid-2020, 81 hospitals were designated as COVID-19 treatment centers. Official statistics reported 17,493 cases and 125 deaths through December 2021, though excess mortality models suggest substantially higher mortality during 2020–2021 (9, 10). While the Tajikistan healthcare system was heavily impacted, there are no official data on healthcare worker mental health outcomes. Data from Central Asia more broadly remain limited; to our knowledge no published studies have examined HCW mental health outcomes during the pandemic in Tajikistan specifically. Understanding the mental health impact of COVID-19 on healthcare workers is critical for strengthening health system resilience. We conducted this study to estimate the prevalence of anxiety and depression and to identify associated occupational and psychosocial factors among healthcare workers in Tajikistan following the first wave of the COVID-19 pandemic.

## Methods

We conducted a retrospective cross-sectional survey of healthcare workers in Khujand city, Sughd region, Tajikistan, between July and August 2022. Khujand, the regional administrative center, was selected as the study site because it houses the only multifunctional referral hospital serving the entire Sughd region. The study included staff from six healthcare facilities (three primary healthcare centers and three hospitals) that provided COVID-19 care between March 2020 and December 2021. Systematic sampling from complete staff rosters was applied consistently across all six facilities stratified by type of worker: every fifth healthcare worker was selected at each site to achieve a target sample size of 400. All selected participants were reached and gave consent to participate.

After obtaining verbal informed consent, face-to-face interviews were conducted using a structured questionnaire that included sociodemographic characteristics, occupational role, working stressors and supports during the pandemic consistent with Job Demands–Resources model, COVID-19 infection history, and psychosocial experiences. Participants were asked to recall their experiences specifically from March 2020 and December 2021. Anxiety and depression were assessed using the Hospital Anxiety and Depression Scale (HADS) (11). We used a cut-off of ≥11 on each subscale to identify cases (moderate and severe); scores of 8–10 were considered borderline (12), given our focus on clinically significant symptom burden in a resource-limited setting. Scores of 8–10 were classified as borderline and are reported descriptively; use of a lower threshold would yield substantially higher prevalence estimates, as noted in the Limitations.

Data were analyzed using R version 4.2.2. Associations with depression and anxiety were examined using multivariable generalized linear mixed-effects models with hospital as a random effect to estimate adjusted prevalence odds ratios (aPORs) and 95% confidence intervals (CIs), accounting for clustering of healthcare workers within hospitals. Ethical approval was obtained from the Ethics Committee of the Ministry of Health and Social Protection of the Population of the Republic of Tajikistan (operating under Resolution No. 1115, June 8, 2018).

This activity was reviewed by U.S. CDC, deemed not research, and was conducted consistent with applicable federal law and U.S. CDC policy. See, e.g., 45 C.F.R. part 46.102(l)(2), 21 C.F.R. part 56; 42 U.S.C. §241(d); 5 U.S.C. §552a; 44 U.S.C. §3501 et seq. No personally identifying information was collected from participants.

## Results

### Study participants

All 400 healthcare workers sampled agreed to participate. Among participants, 69% had moderate to severe depression, 26% had moderate to severe anxiety, and 17% had both conditions. Additionally, 22% had borderline/mild depression and 26% borderline/mild anxiety. The majority of participants were female (82%), nurses (67%), and not in managerial roles (91%). Over half (56%) had been diagnosed with COVID-19. The median years of employment were 11 years [interquartile range (IQR): 5–21].

### Bivariate findings

Female healthcare workers were significantly more likely than males to experience both depression (71% vs. 59%, *p* = 0.04) and anxiety (29% vs. 15%, *p* = 0.02) (Table 1). Nurses had higher prevalence of depression than physicians (77% vs. 56%, *p* < 0.01), but anxiety prevalence did not differ. Those with higher education had elevated anxiety prevalence compared to those with high school education only (31% vs. 18%, *p* < 0.01). Healthcare workers in managerial positions had lower rates of depression (54% vs. 71%, *p* = 0.04).

**Table 1:** Depression and anxiety by socio-demographic characteristics of healthcare workers, Khujand, Tajikistan, 2021.

Characteristics	Total	Had depression		Had anxiety	
	*n* (row %)	*n* (column %)		*n* (column %)	
	*n* = 400	*n* = 277 (69)	*p*	*n* = 104 (26)	*p*
Sex					
Male	74 (18)	44 (59)	^*^	11 (15)	^*^
Female	326 (82)	233 (71)		93 (29)	
Age (in years)					
<35	105 (26)	76 (72)		26 (25)	
35–49	168 (42)	119 (71)		50 (30)	
50+	127 (32)	82 (65)		28 (22)	
Position					
Physician	82 (20)	46 (56)	^**^	18 (22)	
Nurse	269 (67)	207 (77)		73 (27)	
Other	49 (12)	24 (49)		13 (27)	
Education					
Higher or special	243 (61)	160 (66)		76 (31)	^*^
High school	157 (39)	117 (75)		28 (18)	
Years of employment, median (IQR)	11 (5, 21)	10 (5, 21)		12 (5, 23)	
Managerial/leadership position					
Yes	37 (9)	20 (54)	^*^	14 (38)	
No	363 (91)	257 (71)		90 (25)	
History of COVID-19					
Yes	226 (56)	157 (69)		77 (34)	^**^
No	174 (44)	120 (69)		27 (16)	
History of moderate or severe COVID-19					
Yes	33 (8)	28 (85)	^**^	15 (45)	^**^
No or mild COVID-19	367 (92)	249 (68)		89 (24)	
Average hours per day worked					
<12	217 (54)	146 (67)		41 (19)	^**^
12+	183 (46)	131 (72)		63 (34)	
Hours of respirator use					
<3 h or none	362 (90)	257 (71)	^*^	86 (24)	^**^
≥3 h	38 (9.5)	20 (53)		18 (47)	

Values are *n* (%) unless otherwise specified. *p*-values from Pearson’s Chi-squared test; Wilcoxon rank sum test; Fisher’s exact test ^*^p < 0.05; ^**^p < 0.01.

The proportion of healthcare workers with anxiety was higher among those who had COVID-19 vs. those who did not (34% vs. 16%, *p* < 0.01) or who had moderate or severe COVID-19 vs. no or mild COVID-19 (45% vs. 24%, *p* < 0.01). Depression was also higher for those with moderate/severe vs. none or mild COVID-19 (85% vs. 24%, *p* < 0.01). Working ≥12 h daily was associated with increased anxiety compared to those working less than 12 h (34% vs. 19%, *p* < 0.01). Working ≥3 h in a respirator was associated with increased anxiety compared with those working less (47% vs. 24% *p* < 0.01), but lower depression (53% vs. 71% *p* = 0.02).

### Workplace and psychosocial experiences

Multiple workplace stressors showed significant associations with anxiety (Table 2). Poor eating habits at work affected 86% of anxious versus 69% of non-anxious workers (*p* < 0.01). Insufficient sleep due to work was reported by 89% with anxiety compared to 68% without (*p* < 0.01). Lack of time or resources due to work (80% vs. 69%, *p* < 0.01), prolonged PPE discomfort (95% vs. 85%, *p*-value<0.01), panic (66% vs. 43%, *p* < 0.01), crying at work (62% vs. 41%, *p* < 0.01), and fear of work (71% vs. 52%, *p* < 0.01) were all significantly elevated among those with anxiety. Suicidal thoughts were rare overall (2%), but higher among those with anxiety than without anxiety (3.8% vs. 0.7%, *p* < 0.01). Additionally, a higher proportion of people with anxiety than without anxiety reported that their family moved away (27% vs. 40%, *p* < 0.01), loneliness (47% vs. 23%, *p* < 0.01), and social withdrawal (24% vs. 11%, *p* < 0.01).

**Table 2:** Negative experiences in and off work and associations with mental health among healthcare workers, Khujand, Tajikistan, 2021.

Characteristics	All	Anxiety			Depression		
		No	Yes	*p*	No	Yes	*p*
	*N* = 400	*N* = 296	*N* = 104		*N* = 123	*N* = 277	
Negative experiences at work:							
Poor eating habits	292 (73)	203 (69)	89 (86)	^**^	84 (68)	208 (75)	
Insufficient sleep	294 (74)	201 (68)	93 (89)	^**^	85 (69)	209 (75)	
Lack of time or resources	288 (72)	205 (69)	83 (80)	^**^	80 (65)	208 (75)	^*^
PPE discomfort	352 (88)	253 (85)	99 (95)	^**^	106 (86)	246 (89)	
Suicidal thoughts	6 (1.5)	2 (0.7)	4 (3.8)	^**^	4 (3.3)	2 (0.7)	
Panic	196 (49)	127 (43)	69 (66)	^**^	56 (46)	140 (51)	
Crying at work	187 (47)	122 (41)	65 (62)	^**^	57 (46)	130 (47)	
Fear of work	229 (57)	155 (52)	74 (71)	^**^	63 (51)	166 (60)	
Social distancing difficulty	350 (88)	260 (88)	90 (87)		99 (81)	251 (91)	^**^
Trust-building difficulty	290 (72)	219 (74)	71 (68)		77 (63)	213 (77)	^**^
Patient communication difficulty	289 (72)	220 (75)	69 (66)		72 (59)	217 (78)	^**^
Negative experiences outside work:							
Family moved away	79 (20)	51 (17)	28 (27)	^**^	18 (15)	61 (22)	
People avoided me	203 (51)	148 (50)	55 (53)		49 (40)	154 (56)	^**^
Loneliness	117 (29)	68 (23)	49 (47)	^**^	31 (25)	86 (31)	
Social withdrawal	57 (14)	32 (11)	25 (24)	^**^	14 (11)	43 (16)	

PPE, personal protective equipment, Values are *n* (%) unless otherwise specified. *p*-values from Pearson’s Chi-squared test; Fisher’s exact test ^*^p < 0.05; ^**^p < 0.01.

Those with depression experienced had more interpersonal work challenges than those without depression. Difficulty maintaining social distancing at work (91% vs. 81%, *p*-value<0.01), building trust at work (77% vs. 63%, *p* < 0.01), and communicating with patients and their families (78% vs. 59%, *p* < 0.01) were higher among those with depression than those without depression. Additionally, a higher proportion of people with depression than without depression reported that people avoided them during the pandemic (56% vs. 40%, *p* < 0.01) (see Table 3).

**Table 3:** Factors associated with anxiety and depression among healthcare workers, Khujand, Tajikistan, 2021.

Outcome and explanatory variables	POR	95% CI	*p*	aPOR^a^	95% CI	*p*
Outcome: anxiety						
Female (vs. male)	2.3	(1.2–4.8)	^*^	3.2	(1.5–6.6)	^**^
Less than college education (vs. higher)	0.5	(0.3–0.8)	^**^	0.5	(0.3–0.9)	^*^
Worked ≥12 h (vs. < 12 h)	2.3	(1.4–3.6)	^**^	1.9	(1.1–3.2)	^*^
Ever had COVID-19 (vs. not)	2.8	(1.7–4.7)	^**^	2.2	(1.3–3.8)	^**^
Family moved away during pandemic (vs. not)	1.8	(1.0–3.0)	^*^	1.8	(1.0–3.2)	^*^
Had increased fatigue during pandemic (vs. not)	1.8	(1.5–7.9)	^**^	2.4	(1.0–5.7)	^*^
Outcome: depression						
Female (vs. male)	1.7	(1.0–2.9)		0.6	(0.3–1.4)	
Nurse (vs. physician)	2.6	(1.6–4.4)	^**^	3.5	(1.7–7.2)	^**^
Other healthcare workers (vs. physician)	0.8	(0.4–1.5)		0.9	(0.4–1.9)	
Average hour respirator >3 h (vs less)	0.5	(0.2–0.9)		0.4	(0.2–0.9)	^*^
Severe COVID-19 (vs. none/not severe)	2.7	(1.1–8.0)	^**^	4.0	(1.4–11.8)	^**^
People avoided them because of work (vs. not)	1.2	(1.2–2.9)	^*^	1.6	(1.0–2.6)	^*^

POR, prevalence odds ratio; aPOR, adjusted prevalence odds ratio; CI, confidence interval.

a Adjusted for all variables shown within each outcome-specific model. For Outcome: Anxiety, the model included sex, education level, working hours, COVID-19 history, family separation, and fatigue. For outcome: depression, the model included occupational role, respirator use, COVID-19 severity, and social avoidance. ^*^p < 0.05; ^**^p < 0.01.

### Multivariable analysis

In multivariable analyses, anxiety was independently associated with female sex (aPOR = 3.2; 95% CI: 1.5–6.6), working ≥12 h per day during the pandemic (aPOR = 1.9; 95% CI: 1.1–3.2), prior COVID-19 infection (aPOR = 2.2; 95% CI: 1.3–3.8), family separation during the pandemic (aPOR = 1.8; 95% CI: 1.0–3.2), and increased fatigue (aPOR = 2.4; 95% CI: 1.0–5.7). Participants with only high school education had lower odds of anxiety compared with those with higher education (aPOR = 0.5; 95% CI: 0.3–0.9).

Depression was independently associated with being a nurse rather than a physician (aPOR = 3.5; 95% CI: 1.7–7.2), having experienced severe COVID-19 illness (aPOR = 4.0; 95% CI: 1.4–11.8), and experiencing social avoidance related to work (aPOR = 1.6; 95% CI: 1.0–2.6). Working ≥3 h per day in a respirator was associated with lower odds of depression (aPOR = 0.4; 95% CI: 0.2–0.9).

## Discussion

We found high prevalence of depression (69%) and anxiety (26%) among healthcare workers in Khujand, Tajikistan following the first wave of COVID-19. These rates substantially exceed pooled estimates from a review reporting depression prevalence of 21.7% (95% CI, 18.3–25.2%) and anxiety prevalence of 22.1% (95% CI, 18.2–26.3%) among healthcare workers during the pandemic (4). The elevated rates observed here may partly reflect the timing of data collection in 2022, capturing cumulative psychological burden after 2 years of sustained pandemic pressure, in a setting with very limited mental health support infrastructure.

Occupational stressors, particularly extended work hours and fatigue, emerged as critical risk factors for anxiety. The high prevalence of acute emotional reactions (panic, crying, fear of work) among anxious workers suggests anxiety manifests as a direct stress response to overwhelming work demands. During the pandemic in Tajikistan, shifts increased from typical 4–8 h to 6–20 h, with many healthcare workers enduring 14-day rotations in COVID-19 “red zones” with limited rest periods. These findings are consistent with the Job Demands–Resources model in a context where few mental health resources were available to mitigate these stressors (5, 12). Findings also align with international evidence demonstrating that excessive workload significantly impact mental health outcomes among healthcare workers (13).

The finding that working ≥12-h shifts was associated with more than 1.9-fold increased odds of anxiety (aPOR 1.9; 95% CI 1.1–3.2) has direct policy relevance. We recommend that the Tajikistan Ministry of Health consider implementing structured shift rotation systems that cap continuous duty periods, mandate adequate rest intervals between shifts, and establish accessible mental health support points within facilities. Evidence from other healthcare systems suggests that even brief, structured psychological debriefing opportunities and peer support programs embedded in the workplace can meaningfully reduce burnout and anxiety among HCWs (14).

Female sex was associated with more than 3-fold increased odds of anxiety (aPOR 3.2; 95% CI 1.5–6.6). This finding warrants contextual interpretation. In Tajikistan, as in many countries in the region, traditional gender norms place primary home and childcare responsibilities on women. Female healthcare workers on the frontline during the pandemic thus faced a dual burden: managing intensive occupational demands while simultaneously shouldering domestic responsibilities, often with limited social support due to pandemic restrictions. This work–family conflict is a well-established driver of mental health distress among female HCWs (15), and its effects may be particularly pronounced in settings where institutional support for working mothers, such as childcare provisions or flexible scheduling, is limited.

Nursing role was associated with substantially higher odds of depression compared to physician role (aPOR 3.5; 95% CI 1.7–7.2), consistent with findings from studies in other countries (16). Nurses in Tajikistan typically have longer direct patient contact hours, less institutional authority to modify working conditions, and lower average compensation than physicians, which may compound occupational vulnerability. This finding argues for targeted mental health support programs specifically designed for nursing staff.

Working ≥3 h per day in a respirator was paradoxically associated with lower odds of depression (aPOR 0.4; 95% CI 0.2–0.9) despite being associated with higher anxiety in bivariate analysis. This paradoxical finding should be interpreted with caution, as it may reflect confounding by job role or workplace setting rather than a direct protective effect of respirator use. One possible interpretation is that during the severe shortage of respirators in Tajikistan, healthcare workers with consistent access may have felt greater institutional support, potentially reducing the sense of vulnerability that contributes to depression. However, this remains a hypothesis and further research with more granular data on PPE access and job role is needed to disentangle these associations. Studies have consistently shown that adequate protective equipment is essential for healthcare worker mental health (17).

Interpersonal disruptions both within and outside of work emerged as significant factors associated with mental health outcomes. While all healthcare workers experienced high levels of workplace interpersonal challenges (72–88%) and social difficulties outside work (20–51%), the proportion was markedly higher among those with anxiety and depression. Notably, anxiety and depression showed distinct patterns: anxiety was associated with personal isolation experiences (loneliness, family separation, social withdrawal), while depression was uniquely linked to professional relational disruptions (difficulty winning patient trust, communicating with families, maintaining social distance). These findings align with evidence demonstrating that erosion of social and professional connections contributes to mental health distress among healthcare workers facing crisis conditions (13).

These patterns of mental health distress are not unique to the acute pandemic period. Emerging evidence suggests that psychological effects among HCWs may persist well into the post-pandemic era, manifesting as chronic burnout, post-traumatic stress symptoms, and sustained occupational disengagement (18, 19). This underscores the importance of longitudinal follow-up studies in Tajikistan and comparable settings to track trajectories of recovery and to evaluate the effectiveness of any interventions implemented.

### Implications for healthcare systems

Our findings have direct implications for future pandemic preparedness. The clustering of modifiable risk factors—work hours, fatigue, access to personally protective equipment, family separation—suggests multiple intervention points. Studies demonstrate that effective support programs in other countries have included mentoring systems, regular psychological consultations, and tiered support programs (14). These approaches require adaptation to local cultural and social contexts to maximize effectiveness.

### Public health response

The findings from this study prompted immediate action by the Tajikistan Ministry of Health and Social Protection. Mental health trainings were conducted to help healthcare workers and supervisors recognize and manage anxiety and depression. Screening programs were established at healthcare facilities to systematically identify workers experiencing mental health challenges and connect them with appropriate services. Facilities implemented multiple strategies including providing increased access to professional mental health counseling, establishing formal respite programs to reduce continuous work exposure, improving break room access, and adjusting staffing patterns to enable adequate rest periods. These interventions represent critical first steps toward building a more resilient healthcare workforce, though sustained investment and evaluation of program effectiveness will be essential.

### Limitations

This study has limitations. Data were collected from healthcare workers in one urban area, limiting generalizability to rural settings. The retrospective cross-sectional design precludes establishing temporal causality between exposures and outcomes; we cannot determine whether observed mental health symptoms preceded, coincided with, or resulted from the reported occupational exposures. Self-reported data may introduce recall bias, though we minimized this by clearly defining timeframes. Reliance on self-report instruments (HADS) also introduces potential response bias, including social desirability effects. We used a HADS score of ≥11 to define cases, which is more conservative than cut-offs used in other studies which include borderline or mild cases scoring 8–10. This approach may underestimate the true prevalence of anxiety and depression and reduce effect sizes. Importantly, the survey did not assess pre-existing mental health diagnoses or psychiatric treatment history prior to the pandemic. The presence of pre-existing conditions is a significant confounding variable that could affect the interpretation of HADS scores as pandemic-attributable, and future studies should incorporate this screening. Systematic sampling may have excluded workers who were absent on interview days, potentially introducing selection bias. Finally, the survey could not capture the full complexity of mental health experiences.

## Conclusion

Healthcare workers in Tajikistan experienced high rates of depression and anxiety following the COVID-19 pandemic. These findings contribute to the limited evidence on healthcare worker mental health in Central Asia and highlight patterns of mental health distress that may inform future research and preparedness efforts. The strong associations with modifiable factors, such as working hours, fatigue, access to PPE, and social support, point to concrete targets for intervention, and the particularly elevated risk among female healthcare workers and nurses warrants targeted programmatic attention. The immediate public health response, including training, screening, and facility-level interventions, demonstrates the value of evidence-informed action to protect healthcare worker mental health. Sustaining and expanding these programs can help build resilient health systems capable of protecting healthcare workers during future health crises.

## Data Availability

The raw data supporting the conclusions of this article will be made available by the authors, without undue reservation.
